# Association Between Physical Activity and the Risk of Burnout in Health Care Workers: Systematic Review

**DOI:** 10.2196/49772

**Published:** 2024-03-18

**Authors:** Pierpaolo Mincarone, Antonella Bodini, Maria Rosaria Tumolo, Saverio Sabina, Riccardo Colella, Linda Mannini, Eugenio Sabato, Carlo Giacomo Leo

**Affiliations:** 1 Research Unit of Brindisi, Institute for Research on Population and Social Policies, National Research Council Brindisi Italy; 2 MOVE-Mentis s.r.l Cesena Italy; 3 Institute for Applied Mathematics and Information Technologies “Enrico Magenes”, National Research Council Milan Italy; 4 Biological and Environmental Sciences and Technology Department, University of Salento Lecce Italy; 5 Institute of Clinical Physiology, National Research Council Lecce Italy; 6 Innovation Engineering Department, University of Salento Lecce Italy; 7 Respiratory Diseases Unit, “Antonio Perrino” P.O., Local Health Unit “ASL Brindisi” Brindisi Italy

**Keywords:** burnout, Maslach Burnout Inventory, MBI, Copenhagen Burnout Inventory, CBI, Professional Fulfillment Index, PFI, physical activity, health care workers, public health policy

## Abstract

**Background:**

Burnout is a multidimensional psychological syndrome that arises from chronic workplace stress. Health care workers (HCWs), who operate in physically and emotionally exhausting work contexts, constitute a vulnerable group. This, coupled with its subsequent impact on patients and public economic resources, makes burnout a significant public health concern. Various self-care practices have been suggested to have a positive effect on burnout among HCWs. Of these, physical activity stands out for its ability to combine psychological, physiological, and biochemical mechanisms. In fact, it promotes psychological detachment from work and increases self-efficacy by inhibiting neurotransmitters and neuromodulators, increasing endorphin levels, enhancing mitochondrial function, and attenuating the hypothalamic pituitary-adrenal axis response to stress.

**Objective:**

Our objective was to conduct a systematic review of the evidence on the association between physical activity and burnout among HCWs.

**Methods:**

We considered HCWs, physical activity, and burnout, framing them as population, exposure, and outcome, respectively. We searched APA PsycArticles, MEDLINE, and Scopus until July 2022. We extracted relevant data on study design, methods to measure exposure and outcome, and statistical approaches.

**Results:**

Our analysis encompassed 21 independent studies. Although 10% (2/21) of the studies explicitly focused on physical activity, the remaining investigations were exploratory in nature and examined various predictors, including physical activity. The most commonly used questionnaire was the Maslach Burnout Inventory. Owing to the heterogeneity in definitions and cutoffs used, the reported prevalence of burnout varied widely, ranging from 7% to 83%. Heterogeneity was also observed in the measurement tools used to assess physical activity, with objective measures rarely used. In total, 14% (3/21) of the studies used structured questionnaires to assess different types of exercise, whereas most studies (18/21, 86%) only recorded the attainment of a benchmark or reported the frequency, intensity, or duration of exercise. The reported prevalence of physically active HCWs ranged from 44% to 87%. The analyses, through a variety of inferential approaches, indicated that physical activity is often associated with a reduced risk of burnout, particularly in the domains of emotional exhaustion and depersonalization. Furthermore, we compiled and classified a list of factors associated with burnout.

**Conclusions:**

Our comprehensive overview of studies investigating the association between physical activity and burnout in HCWs revealed significant heterogeneity in definitions, measurements, and analyses adopted in the literature. To address this issue, it is crucial to adopt a clear definition of physical activity and make thoughtful choices regarding measurement tools and methodologies for data analysis. Our considerations regarding the measurement of burnout and the comprehensive list of associated factors have the potential to improve future studies aimed at informing decision-makers, thus laying the foundation for more effective management measures to address burnout.

## Introduction

### Background

Burnout is a multidimensional psychological syndrome resulting from chronic workplace stress. It is characterized by feelings of energy depletion or exhaustion, increased mental distance from one’s own job or cynicism, and reduced professional efficacy [1].

Burnout is gaining attention as a major public health concern for the mental health challenges of health care workers (HCWs) [2]. It poses a threat to the quality of care delivery, especially in terms of patient safety [3], and results in high resource consumption to face its consequences [4,5]. This became even more evident during the COVID-19 pandemic [6], which tightened physicians’ work conditions [7,8], significantly increasing the share of physicians and nurses expressing an intention to leave the profession [9,10], and highlighted the frailty of health care systems in emergency management [11]. Although COVID-19 no longer constitutes a public health emergency [12], HCWs continue to be particularly vulnerable to burnout [13,14], with weakened abilities to manage the typical difficulties of care work and increased exposure to emotionally challenging situations [15]. This is especially true for those directly engaged in patient care given the demanding nature of their roles [16]. They often work long hours, experience sleep deprivation, contend with irregular schedules, and are exposed to emotionally challenging situations. In addition, they face the pressure to master a vast body of clinical knowledge [17]. Furthermore, HCWs are at high risk of workplace injuries [18] and assaults [19,20]. These challenges are compounded by common life stressors such as work-home conflicts, educational debts, relationship status, the age of their children, and the employment status of their partners [21]. As reported in a literature analysis [6], burnout can result in various negative outcomes and criticalities for HCWs, including anxiety, depressive disorders, alcohol abuse or dependence, and suicidal ideation. Burned-out workers experience poor mental health even without a clinically diagnosable disorder, as also proved by the fact that, within this group, there are more mental health problems than in most other occupational groups [22].

Within the broad spectrum of possible public health options to address burnout in HCWs, prioritizing measures to promote mental well-being has become paramount [23,24]. This entails addressing cultural factors, particularly those related to stigma; ensuring protected access to mental health care services; and implementing active policies to encourage healthy lifestyles [25,26]. Among these, physical activity is recognized worldwide as a key strategy for promoting mental well-being [27,28] in addition to helping prevent and manage noncommunicable diseases. However, unlike physical health [29], determining the “optimal dose” of physical activity—considering the combination of intensity, duration, and frequency [30]—for mental well-being remains uncertain and dependent on the specific domain of investigation [27].

A preliminary search of previous systematic reviews on the effects of physical activity on burnout was conducted in Scopus, PubMed, and PROSPERO and resulted in 2 different studies. Naczenski et al [31] found that physical activity was an effective strategy to reduce burnout among workers from various sectors. Regarding health professionals specifically, Bischoff et al [32] identified a potential beneficial effect in health professionals of mind-body practices such as yoga or qigong on occupational stress, one of the conditions for burnout [1].

### Objectives

Our study systematically investigated how physical activity was incorporated into studies on burnout among HCWs. Recognizing that strategies created without evidence lead to ineffective programs, wasted resources, and persistently poor health outcomes [33], our ambition was to contribute to the development of evidence-based public health policies.

First, we aimed to provide insights into the reported level of participation in physical activity and the extent of participants at high risk of burnout. We also intended to verify whether a correlation emerges between levels of physical activity and burnout and whether a dose-response association exists.

Our second purpose was to thoroughly assess the quality of the available evidence in terms of collecting, compiling, managing, analyzing, and using health data. This assessment could also offer indications to generate hypotheses for further research to strengthen the body of evidence.

These are essential steps along the road that leads to shaping public health strategies and resource allocation for HCWs’ well-being.

## Methods

As recommended for systematic reviews of association studies [34,35], we adopted the population, exposure, and outcome (PEO) approach by considering physical activity habits as the exposure factor and burnout as the outcome. To ensure accuracy and transparency, we followed the PRISMA (Preferred Reporting Items for Systematic Reviews and Meta-Analyses) [36] and the PRISMA-S (PRISMA literature search extension) [37] guidelines.

### Eligibility Criteria

The eligibility criteria are detailed in Multimedia Appendix 1.

We included studies that examined the association between physical activity and burnout in health personnel directly involved in the provision of care services, such as physicians, nurses, and technicians, with no restrictions on demographics or workplace context. We considered both qualitative and quantitative measures of physical activity as eligible for inclusion. As we were interested in the association between physical activity and burnout, interventional studies were included regardless of the presence of a control group. A validated assessment tool for burnout (a general work-related stress outcome was not of interest) was mandatory for inclusion.

Our search was not limited by geographic context, funding source, or time horizon. We excluded studies in which physical activity was not distinct from other supportive strategies aimed at managing stress or building resilience or from other mindfulness practices. In addition, studies that focused on yoga or qigong were excluded as these are classified as meditation practices with only a light-intensity component of physical activity when performing respiration and poses [38-40].

Following the recommendation by Munn et al [35] to clearly report the exposure or risk factor and how it was measured or identified, we excluded studies that proposed only a simple question on physical activity without any reference to its frequency (eg, a generic “Yes/No” question on practicing “exercise” or even “regular exercise”). Furthermore, we excluded literature reviews, gray literature, conference proceedings, and unpublished material. Finally, we excluded studies that lacked a quantitative evaluation of the association.

### Information Sources

We searched the following electronic databases up to July 2022: MEDLINE through the Ovid platform, APA PsycArticles, and Scopus. Using a “snowballing” approach, we manually screened the reference lists of included articles and conducted systematic citation tracking in Scopus, PubMed, and Google Scholar.

### Search Strategy

In total, 2 authors, CGL and PM, developed search strings using the PEO framework to structure the research question. We selected search terms to identify HCWs, physical activity interventions, and burnout. Regarding burnout, to increase search sensitivity, we also considered terms related to the assessment instruments indicated by Rotenstein et al [41] and those identified in the more recent works by Edú-Valsania et al [42] and Shoman et al [43]. Moreover, to further increase the sensitivity of our search, we used the National Library of Medicine–controlled vocabulary thesaurus (Medical Subject Headings) with entry terms and synonyms. We limited the search to articles published in English. The complete search strategy can be found in Multimedia Appendix 2 [41-43].

Duplicates were removed by PM using an automatic procedure based on PubMed ID and digital object identifier, and this was performed in Microsoft Excel (Microsoft Corp).

### Selection Process

Search results were retrieved from the databases and double screened independently by all the authors. The initial screening was based on the title and abstract using Rayyan (Rayyan Systems Inc) [44] for support. To refine and clarify the eligibility criteria, ensure consistency when applied by different reviewers, and train the team, a pilot phase was carried out on 500 works. The potentially relevant articles were retrieved for full-text screening, and their eligibility was determined as described in the previous step. Any disagreement was resolved through plenary discussion among all the authors until a consensus was reached.

### Data Collection Process

Each selected study was randomly assigned to and independently evaluated by 2 authors to extract relevant data. Any disagreement was addressed as mentioned previously.

### Data Items

We extracted 3 different types of information. First, we recorded the general characteristics, such as aim, context (country, workplace, and period), and population (type of HCW, number, and gender mix). Second, we summarized the methods used to measure burnout, the criteria used to quantitatively summarize the phenomenon in the study population, and a possible definition of severity. When possible, we included the frequency distributions of categorical outcomes and means of total scores with SDs. Third, we noted the methods adopted to measure physical activity, including the assessment of frequency, time, or intensity of activity and the percentage of participants practicing physical activity. Finally, we reported the adopted measures of the association between physical activity and burnout along with its strength (*P* values, odds ratios, and their 95% CIs), eventually derived by AB from published data, and a synthesis of the reported evidence. In addition, factors other than physical activity that are significantly associated with burnout were listed. A first list was drawn from bivariate analyses and reported only variables that were significantly associated (*P*<.05) with burnout. The consideration of even weak associations was hampered by the often incomplete presentation of these analyses. A second list came from multivariable analyses and included all the variables considered in the final (or presented) models regardless of whether physical activity was included in the model itself.

### Synthesis of Evidence

The data are presented in a tabular form.

A narrative approach was adopted to provide an overall summary of the findings of the included studies and their biases, strengths, and limitations, with an in-depth discussion of the causes of heterogeneity.

### Quality

The quality of the studies included in the analysis was assessed using the Joanna Briggs Institute Critical Appraisal Checklist for Cross-Sectional Studies [45]. This checklist consists of 8 items that are rated as “yes,” “no,” “unclear,” or “not applicable.” To further refine our judgment, we added “partial” as a fifth option.

Owing to the eligibility criteria adopted, 2 issues were always rated positively: item 1 (“Were the criteria for inclusion in the sample clearly defined?”), as we only considered studies that clearly reported associations in health personnel directly involved in the provision of care services, and item 4 (“Were objective, standard criteria used for measurement of the condition?”), as we only included studies that used validated questionnaires for burnout.

Item 2 allowed us to register whether a complete description of the participants and setting was provided. We assigned a score of “partial” or “no” if some or all the information was missing.

To assess whether the outcomes were measured in a valid and reliable way (item 7), we verified that the burnout measurement tools were used consistently with the dictates expressed by their developers.

We evaluated the validity and reliability of the exposure measurement (item 3) by positively appraising the use of structured questionnaires (eg, the International Physical Activity Questionnaire–Short Form [IPAQ-SF]) or automatic measurement devices such as pedometers, whereas the use of ad hoc questions to register the frequency or intensity of physical activity was considered a partial achievement.

Concerning the confounding factors, we did not assess how they were considered (item 5) and addressed in the statistical analyses (item 6) in studies exploring multiple wide-scope associations with burnout. On the other hand, we applied these items in studies that focused on a specific exposure factor (either physical activity or another variable of interest). In such cases, the use of statistical modeling, such as multivariable regression analysis, was considered a partial achievement in the absence of an advisable dedicated discussion on confounding factors.

For the appropriateness of the statistical analysis (item 8), a partial quality level was assigned if multivariable regression analysis, though possible, was not conducted or when it was conducted without an adequate method of model selection. Moreover, we assigned a partial level of quality if it was not possible to clearly understand all the details of the analyses because of omissions or results not being clearly reported.

Each study was evaluated for quality by 2 authors, and AB, a statistician, also reviewed items pertaining to statistics.

Discrepancies were resolved through plenary discussion among all authors until a consensus was reached. The quality assessment was not taken into account for eligibility purposes.

## Results

### Selected Studies

**Figure 1:**
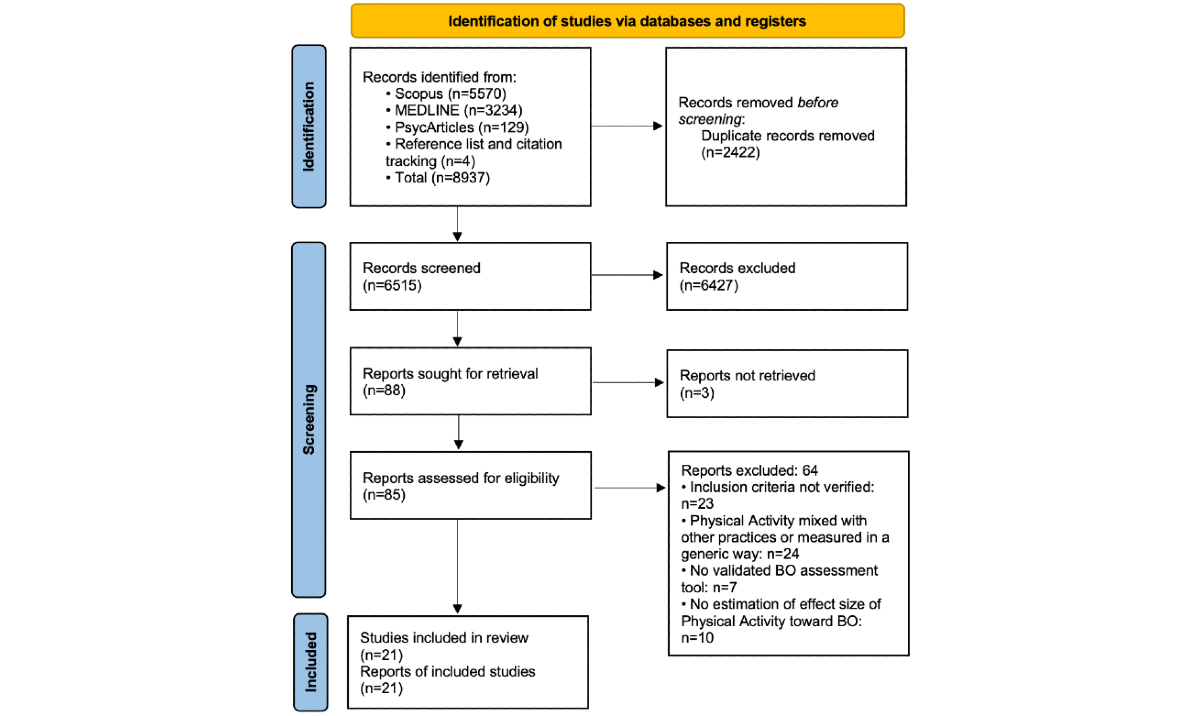
PRISMA (Preferred Reporting Items for Systematic Reviews and Meta-Analyses) 2020 flow diagram. BO: burnout.

A total of 8937 records were identified, and after removing 2422 (27.1%) duplicates (Figure 1), 6515 (72.9%) publications remained following the initial screening. Of these 6515 publications, 6427 (98.65%) were excluded based on the title and abstract. These records were disregarded as they did not address physical activity, burnout, or health care personnel or because they were conference proceedings, reviews, or nonoriginal research (eg, letters or commentaries). Of the 88 studies selected for potential inclusion, 3 (3%) were not retrieved as they were published in journals not accessible through our organizations even after writing to the authors to request the accepted versions of their manuscripts [46-48]. Following full-text screening, 75% (64/85) of the records were excluded. Consequently, 21 independent studies involving 15,782 HCWs were included in this review (see Table 1 for details).

**Table 1:** Characteristics of the included studies.

Study, year	Aim	Country	Period of the survey	Type of HCW^a^	Sample size, N	Sex (female participants), %	Age (y)
Ajab et al [49], 2021	To understand the availability of personal protective equipment and the levels of anxiety, depression, and burnout of HCWs	Asia; United Arab Emirates	From July 2020 to August 2020	Physicians, nurses, allied health care professionals, and laboratory technicians; not specified occupation	1290	78	Mean 38.7 (SD 8.73)^b^
Alvares et al [50], 2020	To assess the prevalence of and factors associated with burnout syndrome	America; Brazil	From November 2011 to June 2013	Nurses and physicians in the ICU^c^	125 nurses and 116 physicians	Nurses: 87.2^d^; physicians: 59.5^d^	Nurses: mean 36.5 (SD 8.2); physicians: mean 38.5 (SD 8.3)
Bin Dahmash et al [51], 2020	To explore the prevalence of burnout and its predictors in radiology residents to minimize burnout rates and improve radiology residents’ well-being	Asia; Saudi Arabia	February 2019	Radiology residents	108	46.3	Mean 27.2 (SD 1.83)^b^
Chokri et al [52], 2021	To determine the prevalence of burnout among health care professionals and investigate the relationship between burnout and sociodemographic characteristics, working systems, and the level of physical activity in health care professionals	Africa; Morocco	February 2021	Nurses, physicians, and health technicians	145	70	Mean 38.3 (SD 9.44)^b^
Portero de la Cruz et al [53], 2020	To estimate the burnout, perceived stress, job satisfaction, coping, and general health levels experienced by nurses working in Spanish emergency departments and analyze the relationship between the sociodemographic, occupational, and psychological variables and the occurrence of burnout syndrome among these professionals	Europe; Spain	From March 2016 to December 2016	Nurses	171	73.1	Mean 47.85 (SD 8.11)
Eckstein et al [54], 2022	To examine how burnout is related to mindfulness, fulfillment, specialty choice, and other lifestyle factors	America; United States	From December 2019 to February 2020	Physicians from different specialties (residents and attending physicians at an academic institution)	60	—^e^	Median 31 (range 25-70)
Feng et al [55], 2018	To determine the prevalence of burnout among ophthalmology residents through a national survey and associate burnout with demographic factors, year in training, practice setting, self-reported workload physical activity, and sleep; in addition, this survey sought to solicit comments from ophthalmology residents regarding factors that they personally felt positively and negatively affected wellness and quality of life	America; United States	From January 17, 2017, to March 18, 2017	Ophthalmology residents from PGY-2^f^ to PGY-4^g^	267	46.1	Mean 29.7 (SD 2.3)
Ghoraishian et al [56], 2022	To evaluate the frequency and factors associated with occupational burnout in orthopedic specialists and residents	Asia; Iran	2019	Orthopedic surgeons and residents	180	5.6	Mean 42.8 (SD 11.17)
Goldberg et al [57], 1996	To measure the degree of burnout among emergency physicians and identify and rank predictive factors	America; United States	During the ACEP^h^ Annual Scientific Assembly years 1992-1995	Emergency physicians	1272	25.7	Mean 39
Hu et al [58], 2021	To investigate the severity of burnout and its associated factors among physicians and nurses in ICUs	Asia; China	From July 25, 2019, to July 30, 2019	Physicians and nurses in ICUs	2411	68.7	Mean 33.5 (SD 5.95)^b^
Lebensohn et al [59], 2013	To study the associations between commonly used indicators of well-being (perceived stress, depression, burnout, and satisfaction with life) and wellness behaviors at the start of family medicine residency	America; United States	At the beginning of PGY-1^i^ in the graduating classes of 2012 and 2013	PGY-1 family medicine (residents)^b^	168	59.9	Median 29
McClafferty et al [60], 2021	To document the concerning state of burnout in early pediatric trainees and examine the potential of the University of Arizona Center for Integrative Medicine PIMR^j,k^ curriculum to provide interventions that address gaps in lifestyle behaviors with recognized association with burnout and how they might be introduced into residency training	America; United States	First trimester of residency in 4 consecutive years from 2012 to 2015	First-year pediatric residents	203	76	Mean 28
Ng et al [61], 2020	To examine the prevalence and severity of burnout and explore the factors (sociodemographic, lifestyle behaviors, and career satisfaction) associated with burnout among medical graduates up to 20 y after graduation	Asia; Hong Kong	From January 29, 2016, to April 15, 2016	Physicians entering medical school	447	43.6	Mean 34.1 (SD 6.0)
Olson et al [62], 2014	To determine the association between achievement of national physical activity guidelines and burnout in internal medicine resident physicians	America; United States	From September 2012 to October 2012	Internal medicine physicians (residents)	76	47.3	Mean 29.2 (SD 2.9)
Panse et al [63], 2020	To assess burnout in plastic surgery residents	Asia; India	From March 2019 to April 2019	Plastic surgery residents	185	28.4	—
Reed et al [64], 2020	To explore the correlations between resident burnout and procedure volume, nonclinical responsibilities, and mindfulness practices along with gathering updated work hour data	America; United States	Not reported	Otolaryngology residents	182	—	—
Shanafelt et al [65], 2012	To evaluate the health habits, routine medical care practices, and personal wellness strategies of American surgeons and explore associations with burnout and quality of life	America; United States	October 2010	Surgeons	7197	14.6	Median 53
Tiwari et al [66], 2020	To investigate the prevalence of burnout among rheumatology practitioners and its associations	America; United States^l^	February 2019	Rheumatology practitioners	128	53.7	Mean 49.9 (SD 12.0)^b^
Vinnikov et al [67], 2019	To assess burnout prevalence in physicians and nurses of a cardiological hospital and ascertain whether smoking, alcohol, and physical activity may predict job-associated burnout	Asia; Kazakhstan	September 2018	Cardiology personnel: physicians, nurses, and technical personnel	259	82	Median 34
Vinnikov et al [68], 2021	To verify the prevalence of occupational burnout in oncology physicians and nurses in a major cancer center and elucidate its predictors to plan future prevention activities	Asia; Kazakhstan	2020 (before the breakout of COVID-19)	Oncology physicians and nurses	256	62	Median 37.5
Yang et al [69], 2018	To measure the prevalence of burnout and resilience levels in transplant nurses, identify any relationships between the 2 concepts, and determine whether demographic factors were associated with burnout in this group	Asia; China	From July 2015 to November 2015	Transplant nurses	536	96.3	Mean 28.40 (SD 4.80)

^a^HCW: health care worker.

^b^Estimated by the authors from grouped data.

^c^ICU: intensive care unit.

^d^Calculated by the authors as, in the paper, the reported percentages were weighted for the unequal probabilities of participant selection.

^e^Not available.

^f^PGY-2: postgraduate at year 2.

^g^PGY-4: postgraduate at year 4.

^h^ACEP: American College of Emergency Physicians.

^i^PGY-1: postgraduate at year 1.

^j^PIMR: Pediatric Integrated Medicine in Residency.

^k^The 10-hour PIMR curriculum is designed in part to help pediatric programs meet new resident well-being requirements. The topics covered include self-nutrition and physical activity, mind-body therapies, dietary supplements, whole systems of medicine, and clinical applications.

^l^The location of the conference where the survey was administered was considered to determine the country.

Although 10% (2/21) of the studies explicitly focused on exploring the association between physical activity and burnout [62,67], the remaining studies investigated the association among several factors, including physical activity. Most of the studies were conducted in America (10/21, 48%) and Asia (9/21, 43%), with only 10% (2/21) of the studies [49,52] being conducted after the spread of COVID-19. In total, 43% (9/21) of the studies [51,54-56,59,60,62-64] focused on residents. All the studies included in this review (21/21, 100%) had a cross-sectional design.

### Demography of Study Populations

The study populations consisted of diverse groups of HCWs with varying proportions of women individuals, ranging from 5.6% to 96.3%. The professional categories represented included physicians, nurses, and technicians from different specialties, which were present in 90% (19/21), 38% (8/21), and 14% (3/21) of the studies, respectively. The sample sizes ranged from 60 to 7197 participants. The mean age varied from 27.2 (SD 1.83) years to 49.9 (SD 12.0) years, with a few studies (5/21, 24%) reporting the median ages, ranging from 29 to 53 years.

### Measurement of Burnout

In terms of burnout measurement, the 22-item Maslach Burnout Inventory (MBI) was the most commonly used questionnaire. Some studies (3/21, 14%) used MBI-related measures based on fewer items [63-65]. In total, 10% (2/21) of the studies used different questionnaires: the Professional Fulfillment Index [54], which is designed to measure physician well-being [70], and the Copenhagen Burnout Inventory [61], which was proposed as an alternative tool to the MBI for measuring burnout [71] (see Multimedia Appendix 3 [49,51-53,55,56,58-63,66-69,72,73] for details).

The severity of the condition in the investigated population was reported in all the studies (21/21, 100%), albeit with differences in terms of the dimensions considered and the criteria used for deriving a summary assessment of burnout across the dimensions and, in some cases, by providing measures of location and scale parameters. Further details are provided in Table 2.

**Table 2:** Burnout levels in the study populations^a^.

Study, year	Measure of burnout (MBI^b^)	Frequency distribution						Location and scale parameters			
		EE^c^	DP^d^	PA^e^	Criteria for burnout	Burnout	EE		DP	PA	Burnout
Chokri et al [52], 2021	MBI; normative data	*65%*^f^, 15%, and 21%	*48%*, 28%, and 23%	*32%*, *25%*, and *38%*	4 classes—high: EE+^g^, DP+, and PA−; moderate: 2 dimensions critical^h^; low: 1 dimension critical; absent: no critical dimension	11%, 41%, 32%, and 17%	Mean 31.03 (SD 15.5)^i^		Mean 9.8 (SD 6.0)^i^	Mean 34.3 (SD 8.0)^i^	N/A^j^
Portero de la Cruz et al [53], 2020	MBI; normative data	*21%*, 19%, and 60%	*43%*, 28%, and 29%	*53%*, *20%*, and *26%*	3 classes—high: EE+, high DP+, and PA−; moderate: if not high and not absent; absent: EE−, DP−, and PA+	8%, 74%, and 18%	Mean 19.15 (SD 12.05)^i^		Mean 9.82 (SD 6.70)^i^	Mean 37.29 (SD 8.92)^i^	N/A
Tiwari et al [66], 2020	MBI; normative data	*38%*, NR^k^, and NR	*31%*, NR, and NR	*NR*, *NR*, and *21%*	Binary classification: positive if EE+ or DP+ and PA−	51%	NR		NR	NR	N/A
Yang et al [69], 2018	MBI; normative data	*37%*, NR, and NR	*31%*, NR, and NR	*NR*, *NR*, and *8%*	Burnout score defined as the sum of the scores in the 3 dimensions^l^	No threshold defined	Mean 24.38 (SD 9.38)		Mean 7.83 (SD 6.46)	Mean 29.38 (SD 8.53)	Mean 61.59 (SD 17.72)
Lebensohn et al [59], 2013	MBI; normative data	*14%*, 28%, and 58%	*24%*, 26%, and 51%	Not considered	3 classes—high: EE+ and DP+; moderate: if not high and not low; low: EE− and DP−	9%, 51%, and 41%	Mean 17.1 (SD 9.5)		Mean 6.38 (SD 4.7)	N/A	N/A
McClafferty et al [60], 2021	MBI; normative data	*20%*, 25%, and 55%	*32%*, 27%, and 42%	NR	3 classes—high: EE+ and DP+; moderate: if not high and not low; low: EE− and DP−	15%, 50%, and 35%	Mean 18.1 (SD 9.0)		Mean 7.4 (SD 4.9)	Mean 29.5 (SD 6.3)	N/A
Olson et al [62], 2014	MBI; normative data	NR, NR, and NR	NR, NR, and NR	Not considered	Binary classification: high if EE+ and DP+	54%	NR		NR	NR	N/A
Bin Dahmash et al [51], 2020	MBI; scoring key	*57%*, NR, and NR	*32%*, NR, and NR	*NR*, *NR*, and *65%*	Binary classification: high if EE+ or DP+ and PA−	24%	Mean 29.0 (SD 11.0)		Mean 9.60 (SD 6.08)	Mean 27.3 (SD 8.55)	N/A
Feng et al [55], 2018	MBI; scoring key	*55%*^i^, 29%^i^, and 17%^i^	*46%*^i^, 25%^i^, and 28%^i^	71%^i^, 18%^i^, and *12%*^i^	Binary classification: high if EE+ or DP+ and PA−	63%	NR		NR	NR	N/A
Hu et al [58], 2021	MBI; scoring key	Physicians: *61%*, 33%, and 6%; nurses: *57%*, 37%, and 7%	Physicians: *37%*, 36%, and 27%; nurses: *31%*, 37%, and 32%	Physicians: *64%*, *16%*, and *20%*; nurses: *66%*, *14%*, and *20%*	Binary classification: high if EE+ or DP+ and PA−	Physicians: 71%; nurses: 68%	NR		NR	NR	N/A
Vinnikov et al [67], 2019	MBI; scoring key	Physicians: *32%*, 29%, and 39%; nurses: *26%*, 27%, and 47%	Physicians: *52%*, 40%, and 8%; nurses: *45%*, 37%, and 18%	Physicians: 16%, 16%, and *69%*; nurses: 32%, 22%, and *46%*	Not considered	N/A	Physicians: median 19 (IQR 15.8); nurses: median 18 (IQR 17)		Physicians: mean 14.1 (SD 6.4); nurses: median 12 (IQR 9)	Physicians: median 41 (IQR 9); nurses: median 38 (IQR 17)	N/A
Vinnikov et al [68], 2021	MBI; scoring key	Reported data were not consistent with the reported categorization	Reported data were not consistent with the reported categorization	Reported data were not consistent with the reported categorization	Not considered	N/A	Median 26 (IQR 19)		Median 15 (IQR 10)	Median 29 (IQR 15.8)	N/A
Ajab et al [49], 2021	MBI; Ajab et al [49]	*13%*, 40%, and 47%	*4%*, 23%, and 73%	*70%*, *29%*, and *1%*	Not considered	N/A	NR		NR	NR	N/A
Ghoraishian et al [56], 2022	MBI; Ghoraishian et al [56]	*27%*, 29%, and 43%	*16%*, 19%, and 65%	*40%*, *23%*, and *37%*	4 classes—severe: EE+, DP+, and PA−; moderate: 2 dimensions critical; mild: 1 dimension critical; absent: no critical dimension	7%, 16%, 27%, and 50%	NR		NR	NR	N/A
Panse et al [63], 2020	9-item MBI (aMBI^m^) [74]	Moderate to severe: *64%*; no to low: 36.2%	Moderate to severe: *26%*; no to low: 74.1%	Moderate to severe: *91%*; no to low: *9%*	Burnout score defined as the sum of the scores in EE and DP and classified as binary (cutoff of ≥19; no reference provided for the validation of this cutoff)	49%	NR		NR	NR	NR
Goldberg et al [57], 1996	MBI	Not considered	Not considered	Not considered	Binary classification: moderate to high levels of burnout according to the Golembiewski classification^n^	61%	Mean 23.31 (SD 8.55)		Mean 20.70 (SD 8.49)	Mean 24.72 (SD 9.17)	NR
Alvares et al [50], 2020	MBI	Physicians: *27%*, NR, and NR; nurses: *31%*, NR, and NR	Physicians: *7%*, NR, and NR; nurses: *6%*, NR, and NR	Physicians: *NR*, *NR*, and *9%*; nurses: *NR*, *NR*, and *12%*	Two binary classifications: (1) critical values in all the dimensions and (2) critical values in at least one dimension	Physicians: (1) 1% and (2) 34%; nurses: (1) 0% and (2) 39%	NR		NR	NR	N/A
Shanafelt et al [65], 2012	2 single-item measures adapted from the MBI [75]^o^	EE reported at least weekly: 23%	DP reported at least weekly: 15%	N/A	Binary classification^i^: high if EE+ or DP+	27%	N/A		N/A	N/A	N/A
Reed et al [64], 2020	1 single question [76]^p^	N/A	N/A	N/A	N/A	50%	N/A		N/A	N/A	N/A

^a^Frequency distribution from *high* to *low*. Independent of the way the authors reported the frequency distribution for PA, in accordance with Maslach et al [72], we considered low PA as a condition characterizing burnout. The numbers in the frequency distribution are rounded to percentage units.

^b^MBI: Maslach Burnout Inventory. In total, 3 categories were defined: emotional exhaustion, depersonalization, and PA. Most recent edition: Maslach et al [77].

^c^EE: emotional exhaustion.

^d^DP: depersonalization.

^e^PA: personal accomplishment.

^f^Italics indicate critical values for burnout.

^g^The burnout dimension is in the *high* range (+) or *low* range (−).

^h^When referring to MBI and not otherwise specified, critical levels in burnout dimensions mean *high* in EE and DP and *low* in PA.

^i^Authors’ interpretation.

^j^N/A: not applicable.

^k^NR: not reported.

^l^The proposed burnout score does not consider that PA should be interpreted in the opposite direction from EE and DP as a low degree of burnout is reflected in high scores on PA [72].

^m^aMBI: abbreviated Maslach Burnout Inventory.

^n^Raw data from the MBI used to divide the burnout process into 8 phases, with phases I-III representing a low degree and phases IV-V and VI-VIII representing moderate and high degrees, respectively [78].

^o^MBI adapted.

^p^MiniZ (adapted).

In two studies, authors adopted tools not referable to BMI. Ng et al [61] adopted the Copenhagen Burnout Inventory [71], which comprises 3 categories: personal, physical, and psychological exhaustion, work-related physical and psychological exhaustion, and patient-related physical and psychological exhaustion. The reported frequency distribution among study participants is 63%, 56%, and 35% respectively. No specific criteria were defined for burnout.

Eckstein et al [54] used the Professional Fulfillment Index [70], which consists of three response categories: Professional Fulfillment (PF), Interpersonal Disengagement, and Work Exhaustion (WE), employing 5-point Likert scales (“not at all true” to “completely true” for PF items and “not at all” to “extremely” for WE and Interpersonal Disengagement items). All responses are scored from 0 to 4. However, in the considered study, the PF domain was not taken into account. A binary classification (presence vs absence of burnout) is adopted according to the following criterion: the average score of Interpersonal Disengagement and WE ≥1.33 [70]. Participants meeting this criterion for burnout comprise 38%. Regarding Location and scale parameters, the median score for WE, Interpersonal Disengagement, and burnout is respectively 1.50, 0.83, and 1.00.

With due caution regarding the aforementioned heterogeneity of definitions and cutoffs, the percentage of participants classified as burned out varied from 7% to 83%.

### Measurement of Physical Activity

None of the included studies used objective measurement tools, such as pedometers, to assess physical activity (Table 3).

Structured questionnaires that distinguished among different typologies of physical activity or ad hoc items were considered to investigate the habits of HCWs. The IPAQ-SF, the Ricci-Gagnon scale, and the Arizona Lifestyle Inventory were used in the studies by Olson et al [62], Chokri et al [52], and McClafferty et al [60], respectively. The IPAQ-SF was the only validated tool used, although it tends to overestimate actual physical activity levels [84]. A total of 33% (7/21) of the studies [50,53,61,63,67-69] used an ad hoc question to assess the achievement of a given benchmark, with a threshold frequency ranging from 1 day every 2 weeks [63] to every day [53]. In the remaining studies, physical exercise was measured in terms of frequency and intensity [65], frequency and time [57], only frequency [49,51,54-56,58,59,66], or only time [64]. When it was possible to report a distribution of frequencies or times, we considered the lowest category that did not indicate “no physical activity at all.” Whenever possible, we adopted the most widely used benchmark of physical exercise of “at least 1 day per week” to define physically active participants. Using this criterion, we found that the percentage of active workers ranged from 44% to 82% for residents and from 46% to 87% for the other categories.

**Table 3:** Level of physical activity in the study populations.

Study, year	Method used to measure physical activity	Percentage of HCWs^a^ practicing physical activity	
		Criterion for prevalence	Prevalence, %
Olson et al [62], 2014	IPAQ-SF^b^ [79]	Compliance with the DHHS^c^ benchmark: ≥150 min/wk [80]	59
Chokri et al [52], 2021	Ricci-Gagnon scale [81]	Not inactive	77
McClafferty et al [60], 2021	Arizona Lifestyle Inventory [82]	≥1 d/wk for ≥30 min/session of moderate physical activity	85
Portero de la Cruz et al [53], 2020	Question assessing a benchmark achievement	7 d/wk	49
Ng et al [61], 2020	Question assessing a benchmark achievement	Performing regular exercise: ≥5 d/wk for ≥10 min/session of any vigorous or moderate physical activities	76
Vinnikov et al [67], 2019	Question assessing a benchmark achievement	Performing regular exercise: ≥3 d/wk for ≥40 min/session of any off-work physical activity	34
Vinnikov et al [68], 2021	Question assessing a benchmark achievement	Performing regular exercise: ≥3 d/wk of any physical activity	19
Alvares et al [50], 2020	Question assessing a benchmark achievement	Performing regular exercise: ≥3 d/wk of any physical activity	Nurses: 16^d^; physicians: 51.7^d^
Yang et al [69], 2018	Question assessing a benchmark achievement	≥1 d/wk	46
Panse et al [63], 2020	Question assessing a benchmark achievement	≥1 d for 2 wk	35
Shanafelt et al [65], 2012	Frequency and intensity	>30 min/wk (moderately intense aerobic exercise)	≥75
Shanafelt et al [65], 2012	Frequency and intensity	>30 min/wk (vigorously intense aerobic exercise)	55
Shanafelt et al [65], 2012	Frequency and intensity	Compliance with the CDC^e^ recommendation for aerobic exercise and muscle strength training [83]	37
Goldberg et al [57], 1996	Frequency and time	≥1 d/wk, ≥10 min/session	≥78^f^; ≥79^f^
Feng et al [55], 2018	Frequency	Compliance with the DHHS benchmark: ≥150 min/wk [80]	35.2
Ajab et al [49], 2021	Frequency	≥1 d/wk	61
Bin Dahmash et al [51], 2020	Frequency	≥1 d/wk	44
Eckstein et al [54], 2022	Frequency	≥1 d/wk	≥78^f^
Hu et al [58], 2021	Frequency	≥1 d/wk	56
Lebensohn et al [59], 2013	Frequency	≥1 d/wk	79
Tiwari et al [66], 2020	Frequency	≥1 d/wk	87
Ghoraishian et al [56], 2022	Frequency	A clear cutoff was not indicated	N/A^g^
Reed et al [64], 2020	Time	A clear cutoff was not indicated	N/A

^a^HCW: health care worker.

^b^IPAQ-SF: International Physical Activity Questionnaire–Short Form.

^c^DHHS: US Department of Health and Human Services.

^d^Calculated by the authors as the percentages reported in the paper were weighted for the unequal probabilities of participant selection.

^e^CDC: Centers for Disease Control and Prevention.

^f^Authors’ interpretation.

^g^N/A: not applicable.

### Methods of Association Assessment

Bivariate associations between physical activity and burnout were assessed using usual methods. Table 4 provides further details. All studies except those by Olson et al [62], Vinnikov et al [67], Ghoraishian et al [56], Feng et al [55], and Tiwari et al [66] also conducted a multivariable regression analysis. In all 5 cases, a multivariable analysis could have been conducted to obtain adjusted odds ratios. Logistic and linear regression were equally used in 43% (9/21) and 33% (7/21) of the studies, respectively.

**Table 4:** Association between physical activity and burnout.

Study, year	Data analysis method	Bivariate analysis	Multivariable regression analysis
Ajab et al [49], 2021	Bivariate analysis and multivariable linear regression	Low levels of EE^a^ and DP^b^ were significantly more frequent among HCWs^c^ who performed physical activity almost every day than among HCWs who were not physically active the previous week (*P*<.001). High levels of personal accomplishment were significantly more frequent among HCWs who performed physical activity almost every day or every day than among HCWs not physically active the previous week (*P*<.001)	Not clearly reported and not coherent with the statistical methods declared
Alvares et al [50], 2020	Chi-square and Fisher exact tests and multivariable logistic regression	Nurses: participants who did not exercise >3 d/wk^d^ were at higher risk of high levels^e^ of EE (OR^f^ 7.36, 95% CI 1.14-47.32) and at lower risk of high levels^e^ of DP (OR 0.05, 95% CI 0.004-0.61); physicians: crude ORs were not significant	Nurses: adjusted for other covariates, participants who did not exercise >3 d/wk^d^ were at higher risk of high levels of EE (OR 11.01, 95% CI 2.73-44.39) and at lower risk of high levels of DP (OR 0.07, 95% CI 0.007-0.79)
Bin Dahmash et al [51], 2020	Univariable and multivariable logistic regression	Participants who exercised ≥1 d/wk were significantly less at risk of having high DP (OR 0.33, 95% CI 0.13-0.78), low personal accomplishment (OR 0.43, 95% CI 0.19-0.97), or high burnout (OR 0.29, 95% CI 0.10-0.77; *P*=.01) than those who never exercised	Participants who exercised ≥1 d/wk were significantly less at risk of having high DP (aOR^g^ 0.38, 95% CI 0.15-1; *P*=.04) than those who never exercised
Chokri et al [52], 2021	Chi-square test and multivariable linear regression	Degree of physical activity was not significantly associated with degree of EE (*P*=.86); it was weakly associated with DP and personal accomplishment (*P=*.09 and *P*=.08, respectively)	Physical activity not included in the model
Portero de la Cruz et al [53], 2020	Bivariate analysis and univariable and multivariable linear regression	Those who did not take part in daily physical exercise had higher mean DP (*P*=.005) scores. There were no statistically significant differences in mean EE (*P*=.09) and mean personal accomplishment (*P*=.48) according to daily physical exercise	Adjusted for other covariates, the lack of daily physical activity was a significant predictor of higher D*P* values
Eckstein et al [54], 2022	Univariable and multivariable logistic regression	Frequency of exercise not significantly associated with burnout	Physical activity not included in the model
Feng et al [55], 2018	Bivariate analyses	The probability of low EE was significantly higher in participants who engaged in physical activity ≥150 min/wk (*P*=.02). No association was found with levels of DP (*P*=.32), personal accomplishment (*P*=.29), and burnout (*P*=.13)	NP^h^
Ghoraishian et al [56], 2022	Chi-square test and univariable logistic regression	Participants who exercised ≤1 h/wk were at higher risk of burnout than those who exercised >1 h/wk (OR 2.3, 95% CI 1.24-4.48)	NP
Goldberg et al [57], 1996	Chi-square test and multivariable logistic regression	Results of association analysis were inconsistent with the data.	Low levels of exercise were significantly associated with burnout
Hu et al [58], 2021	Chi-square test and multivariable logistic regression	Not reported in the paper	Participants who exercised ≥1 d/wk were significantly at lower risk of burnout (once a week: OR 0.66, 95% CI 0.45-0.95; every 2 or 3 d: OR 0.56, 95% CI 0.39-0.80; every day: OR 0.52, 95% CI 0.36-0.75) than those who exercised less frequently or never
Lebensohn et al [59], 2013	ANOVA and multivariable linear regression	Physical activity was not significantly associated with burnout	More frequent physical activity was a significant adjusted predictor of lower values of both EE and DP
McClafferty et al [60], 2021	ANOVA and multivariable linear regression	Among individuals at high risk of burnout, the frequency of physical activity was lower than the group mean, whereas among individuals at low or moderate risk, the frequency was higher (*P*=.10)	Adjusted for other covariates, a higher frequency of exercise was a significant predictor of a higher score on personal accomplishment
Ng et al [61], 2020	Univariable and multivariable linear regression	Practicing regular exercise significantly reduced CBI^i^-PeE^j^ (slope: −9.882; *P*<.001) and CBI-PaE^k^ (slope: −6.932; *P*=.004). It was not correlated with CBI-WrE^l^	Practicing regular exercise was a significant adjusted predictor of lower values of CBI-PeE and CBI-PaE
Olson et al [62], 2014	Chi-square test and univariable logistic regression	Participants compliant with DHHS^m^ guidelines were significantly less at risk of having high subscores in burnout (OR 0.38, 95% CI 0.147-0.99) than noncompliant HCWs	NP
Panse et al [63], 2020	Univariable and multivariable logistic regression	Participants who performed any physical activity for fitness at least once in 2 weeks were significantly less at risk of high values of burnout (OR 0.41, 95% CI 0.22-0.77) than those who did not perform physical activity	Physical activity was not significantly associated with burnout
Reed et al [64], 2020	Chi-square test, ANOVA, and multivariable logistic regression	Results of association analysis were inconsistent with the data	Physical activity was not significantly associated with burnout
Shanafelt et al [65], 2012	Multivariable logistic regression	NP	Compliance with CDC^n^ aerobic exercise and muscle strength training recommendations was not independently associated with burnout
Tiwari et al [66], 2020	Univariable logistic regression	Participants who lacked exercise at least 1 d/wk were at higher risk of burnout^o^ (OR 5.00, 95% CI 1.3-18.5) than participants who exercised	NP
Vinnikov et al [67], 2019	Bivariate analysis	Physical activity was not significantly associated with any MBI^p^ dimension	NP
Vinnikov et al [68], 2021	Bivariate analysis and multivariable logistic regression	Participants who did not regularly exercise were at higher risk of high EE (OR 5.02^d^, 95% CI 2.25-12.42) and high DP (OR 2.37^d^, 95% CI 1.20-4.74) than those who regularly exercised	Participants who did not regularly exercise ≥3 d/wk were significantly more at risk of having high EE (aOR 9.91, 95% CI 2.92-27.2) than the other participants
Yang et al [69], 2018	Multivariable linear regression	NP	Adjusted for other covariates, exercising every week was a significant predictor of lower values of EE and burnout^q^. It was also a significant predictor of lower values of personal accomplishment

^a^EE: emotional exhaustion.

^b^DP: depersonalization.

^c^HCW: health care worker.

^d^Derived by the authors.

^e^Participants with moderate burnout in the dimension were excluded from the analysis.

^f^OR: odds ratio.

^g^aOR: adjusted OR.

^h^NP: not performed.

^i^CBI: Copenhagen Burnout Inventory. In total, 3 categories were defined: personal physical and psychological exhaustion, work-related physical and psychological exhaustion, and patient-related physical and psychological exhaustion.

^j^PeE: personal physical and psychological exhaustion.

^k^PaE: patient-related physical and psychological exhaustion.

^l^WrE: work-related physical and psychological exhaustion.

^m^DHHS: US Department of Health and Human Services. Its guidelines [80] set a benchmark of 150 minutes per week of physical activity.

^n^CDC: Centers for Disease Control and Prevention. It set recommendations for aerobic exercise and muscle strength training [83].

^o^The definition of burnout adopted in the logistic regression was not clear.

^p^MBI: Maslach Burnout Inventory.

^q^The result was reported for completeness even though we believe that the burnout score was meaningless as it did not consider that personal accomplishment should be interpreted in the opposite direction from EE and DP.

### Existence and Degree of Association

Table 4 also presents the evidence of the association between physical activity and burnout. Owing to the high heterogeneity of the studies, a direct comparison of the results was not feasible. As previously mentioned, the 2 sources of heterogeneity were the diverse and sometimes vague definitions of physical activity used in the studies and the variations in the definitions and cutoffs for measuring burnout.

A total of 14% (3/21) of the studies [49,57,64] reported a few results that were not consistent with the data, and in one case [58], the authors referred to supplementary material that was not available on the journal web page. In total, 80% (4/5) [55,56,62,66] of the studies that presented only bivariate analyses indicated that a lack of physical activity was associated with high values of at least one component of the MBI. Similar results were reported by half (8/16, 50%) of the other studies that presented bivariate analyses regardless of the measure of burnout adopted.

In studies in which bivariate analysis was preparatory to multivariable regression analysis [50-54,58,61,63,68], physical activity may not have emerged as a predictor in the multivariable models [51,52,54,63,68]. This is sometimes due to the lack of a significant (albeit weak) association already in the bivariate analyses [52,54]. However, this result is also influenced by the different approaches to variable inclusion and selection. When conducting a bivariate analysis as a preliminary step for variable selection in the multivariable model, a less stringent significance criterion than *P*<.05 should be considered. Variables that show weak individual associations can become important predictors when considered jointly (eg, the study by Hosmer and Lemeshow [85]). Unfortunately, the selected studies used very different criteria, ranging from *P*<.05 for each variable to no preselection at all. In addition, methods for model selection were not always applied or clearly stated.

In cases in which physical activity was found to be a significant predictor in multivariate analyses, a protective effect was observed, especially against emotional exhaustion [50,59,61,68,69] and depersonalization [50,51,53,59]. This held true across a range of activity frequencies, from as little as 1 session per week to daily engagement.

For the sake of completeness, factors other than physical activity that showed a statistically significant association with burnout have been listed in 2 separate tables: one for bivariate analyses and the other for multivariable analysis (Tables S1 and S2 in Multimedia Appendix 4). The predictors were grouped by topic (demography, health conditions, lifestyle, personal attitude, work-life balance, work organization and environment, work profile, and self-perception at work) and ordered within each class according to their frequency.

### Quality Evaluation

The risk of bias was assessed using the Joanna Briggs Institute tool, and the details are presented in Multimedia Appendix 5 [45,49-69].

All the studies provided detailed descriptions of participants and settings, with the exception of the studies by Eckstein et al [54], in which gender specifications were missing, and Reed et al [64], in which the period of the survey, gender mix, and age were not reported.

Regarding the assessment of physical activity, only a minority of studies (3/21, 14%) [52,60,62] adopted structured questionnaires and, therefore, received a positive evaluation. Most studies (18/21, 86%) only partially fulfilled this criterion, either relying on simple ad hoc questions to assess the activity frequency, time, or intensity or referring to meeting a threshold of activity. This limitation was observed even in studies that claimed to have a specific focus on physical activity [55,67].

Confounding was never explicitly addressed. As mentioned in the Methods section, we applied the dedicated items to the 14% (3/21) of studies that focused on a specific exposure [62,67,69]. All these papers touched on this issue through their conducted analyses and, therefore, obtained a “partial” rating for item 5. Of these studies, 33% (1/3) conducted only a bivariate analysis and, thus, did not comply with item 6 [62].

As for the use of the MBI, only a few studies (9/21, 43%) received a positive evaluation for item 7. Contrary to the explicit indications of the MBI developers [86], the authors of most studies (11/21, 52%) [50,51,55-58,62,63,66-68] adopted a categorical classification of participants based on burnout dimensions for descriptive as well as inferential purposes. Moreover, Yang et al [69], although correctly adopting the score to assess each MBI dimension, defined and adopted an overall burnout score as the raw sum of the scores in each dimension (as reported in Table 4, even without considering that personal accomplishment should be interpreted in the opposite direction from emotional exhaustion and depersonalization). In all these cases except the studies by Ghoraishian et al [56] and Panse et al [63], we assigned a partial achievement of the item. Indeed, these 2 studies were ranked with a “no” as they did not even provide the rationale behind their choice of ranges of scores in each dimension. Except for 14% (3/21) of the studies—Ajab et al [49] and Vinnikov et al [67,68]—all the other studies referring to the MBI or related multi-item measures introduced a definition of overall burnout. Despite this, studies that also adopted the total score of the burnout dimensions in their inferential analyses [52,53,59,60] were ranked with a “yes.” Ng et al [61] and Eckstein et al [54] were classified as “yes” as they correctly adopted the measurement tools—Copenhagen Burnout Inventory [73] and Professional Fulfillment Index [54], respectively.

Regarding item 8, statistical analysis was not considered appropriate in 10% (2/21) of the studies [50,58], in which the significant differences between physicians and nurses in the dimensions of the MBI were not adequately handled in the multivariable analysis. The analyses conducted in another 14% (3/21) of the studies [49,56,64] were rated as unclear either because the presented results were not coherent with the declared statistical methods [64] or because of unexplained methods [49,56]. Goldberg et al [57], Bin Dahmash et al [51], Shanafelt et al [65], Vinnikov et al [68], Portero de la Cruz et al [53], and Chokri et al [52] clearly explained their statistical methods and conducted comprehensive analyses, receiving a positive quality assessment. In the other cases, a better statistical methodology could have been applied by conducting a multivariable analysis, considering more suitable criteria to include the explanatory factors in the multivariable analysis, or again by applying model selection methods to obtain more parsimonious and general models.

## Discussion

### Principal Findings

The COVID-19 pandemic has taken a heavy toll on HCWs in terms of physical and mental health, and the long-term effects of the pandemic will further increase the burden of HCWs’ mental health disorders and burnout [87,88]. It has been argued that addressing burnout could serve as a nonstigmatized and systemic approach to address a long-standing issue in medicine through mental health initiatives, whether prevention oriented or treatment focused [89].

In addition to the personal consequences for HCWs experiencing burnout, it is important to consider its impact on patient care and resource consumption. Emotionally and physically healthy HCWs are among the most relevant factors influencing health care service quality [90]. All these considerations emphasize the importance of implementing strategies to prevent and manage burnout at an individual, organizational, and cultural level. The literature on burnout management primarily presents fragmented solutions that are infrequently tested in practice. These solutions often align with 1 of 2 predominant lines of intervention: one emphasizes strengthening individual capabilities to navigate the inherent challenges of health care work, whereas the other acknowledges work organization as a contributing factor and attempts to intervene at that level. However, there is a growing realization that individual and organizational well-being are intricately interconnected, thus necessitating systemic solutions. These comprehensive approaches encompass organizational interventions, instilling a culture of well-being in the workplace, and integrating well-being into health care education for true efficacy [24,91]. A recent meta-analysis of 20 controlled trials found that the most effective existing interventions for reducing burnout were those targeting multiple facets of well-being [92]. Regrettably, these systemic solutions are complex and often come at a significant cost [93-95]; therefore, it is particularly relevant for research to precisely identify the characteristics that specific interventions must have to succeed. This review focused on physical activity as an effective factor in fostering a culture of well-being among HCWs, which is crucial for tackling the physical and mental consequences of work-related stress [24]. Physical activity has enormous potential to mitigate the physical and mental impacts of work-related stress [96,97]. Indeed, it is suggested that it facilitates psychological detachment from work and enhance self-efficacy [53], providing an opportunity to divert attention from stressful thoughts [50]. It has also been shown that moderate-intensity exercise training programs improve feelings of vigor, energy, and vitality [98]. In particular, greater effects occurred when combining resistance exercise with aerobic exercise compared with aerobic exercise alone [98]. This finding is consistent with the World Health Organization recommendations for physical activity, which underlie the importance for adults of regularly performing both aerobic and muscle-strengthening activities to support health, including mental health outcomes [96]. With this review, we aimed to systematically assess the strength of the evidence and, eventually, the dose-response association between physical activity and burnout in a physically and emotionally exhausting work environment. However, it is essential to note that all studies included in our review had a cross-sectional design. As is well known, randomized controlled trials with a longitudinal perspective are the gold standard to highlight any potential cause-effect relationship between an exposure and an outcome. Nevertheless, the cross-sectional approach is useful in highlighting potential relationships between burnout and related factors, aiding in the identification of a multiplicity of risk factors and mitigation strategies, as the complexity of the phenomenon requires. This is particularly true in our case as most of the included studies (19/21, 90%) were exploratory investigations that examined various aspects potentially related to burnout rather than focusing specifically on physical activity. As shown in Tables S1 and S2 in Multimedia Appendix 4, a wide range of variables were considered in bivariate analyses (74 issues) and included in multivariable models (58 issues), reflecting the interplay between burnout and demographic characteristics, health, lifestyle, personal attitude, self-perception at work, work organization and environment, job profile, and work-life balance.

As mentioned previously, physical activity is considered a potential tool to cope with distress, and the PEO approach adopted in this study was specifically structured in this direction. However, it is important to recognize the possibility that high levels of burnout negatively influence the level of physical activity [99]. According to the study by Olson et al [62], the “lack of energy” among burned-out residents suggests that high levels of burnout lead HCWs to reduce the time dedicated to leisure activities, including physical exercise. Stults-Kolehmainen and Sinha [99] found a similar result in the literature, stating that stress hinders individuals from being more physically active and has a negative influence on other health behaviors, including smoking, alcohol, and drug use. We suggest that future studies include sections dedicated to exploring the reasons for individuals’ inability to meet their desired level of physical activity. Factors such as time constraints, lack of interest, or underlying health conditions should also be accounted for. The Barriers to Being Active Quiz developed by the US Centers for Disease Control and Prevention [100] is an example of a tool that may support the exploration of the dynamic interactions among personal, socioenvironmental, and behavioral factors, serving as a knowledge base for promoting more active and healthy lifestyles.

This study confirms that the dose-response relationship between physical activity and psychological well-being and health-related quality of life is far from being established. Significant mental health benefits could be achieved even at physical activity levels below the public health recommendations [101,102]. This also emerged from our study and has direct implications on healthy lifestyle recommendations, especially for inactive HCWs for whom incorporating brief bouts of physical activity into daily activities may be a more realistically achievable goal than meeting the guideline-recommended physical activity levels. This can be crucial to promote physical activity and, therefore, trigger a virtuous circle with benefits for burnout. In addition, it may have broader implications for the general population as there are indications that physicians’ involvement in physical activity is linked to their propensity to advise patients on the advantages of exercise [103,104]. Recognizing that any physical activity is better than none and considering engagement in physical activity as a modifiable behavior, adopting a strategy of gradually increasing activity through small habit changes is deemed effective for establishing a consistent exercise routine. This approach can be facilitated by the use of activity trackers [105,106].

This systematic review highlighted another general challenge in quantifying the strength of the physical activity–burnout association and establishing the dose-response curve, primarily as various methods were used to detect burnout. Different self-reporting tools are indeed available grounded on different theoretical bases [42]. Most of the studies in our review (19/21, 90%) proposed the 22-item MBI or MBI-related questionnaires (Table 2), confirming the substantial monopoly of this tool in burnout research. All these studies fell into some form of misuse of the index. The 3-factor structure of the MBI implies, on the one hand, that each dimension must be treated separately and, on the other hand, that none of them should be ignored. In contrast, in some studies where it would have made sense (3/17, 18%) [59,60,62], personal accomplishment was excluded from the analysis. This practice is not uncommon as several authors consider personal accomplishment not as a reaction to stressful situations but rather as a personality trait or coping resource and, therefore, as not contributing to the comprehensive concept of burnout [107]. Another misuse of the MBI is its diagnostic application, which erroneously considers the MBI dimensions as symptoms of burnout [71]. The 7-point scale (from *Never* to *Every day*) used to report the feelings experienced by the respondents was intended by Maslach and the coauthors of the tool as an operational simplification of the measurement of a dynamic phenomenon evolving continuously over time rather than as the assessment of a dichotomous condition (absent or present) defined through an arbitrary cutoff. Considering MBI scores for diagnostic purposes would inevitably invoke the wrong concept of burnout as a disease or disability, ignoring decades of research and the recent statement from the World Health Organization [1]. The MBI was not designed as a diagnostic tool [86], and the cutoff scores established to classify people at low, moderate, and high levels of burnout were “intended primarily as feedback for individual respondents.” These scores were published up to the third edition of the MBI Manual [72] accompanied, however, by a strong recommendation to use the original total scores for any statistical analysis. In the fourth edition released in 2018 [77], the categorization was finally removed. Therefore, it is surprising that, despite the extraordinary diffusion of the MBI, there was a failure to implement the correct instructions for its use. In fact, all studies except the one by Goldberg et al [57] considered some classification of the severity (sometimes referred to as *risk*) of burnout in each of the 3 MBI dimensions also for inferential purposes. Furthermore, a variety of classification criteria, even when taken from the same reference [72], and algorithms for combining dimensions into a single overall burnout indicator, typically a high score in at least one dimension and sometimes in all dimensions (reversing personal accomplishment, if considered), further increased the heterogeneity of the analyses. These methodological considerations become even more crucial in future research, particularly now that apps providing burnout self-diagnosis are available and transparency is needed in the adopted assessing algorithms [108].

Moving on to the evidence on the association between physical activity and burnout that emerged from this review, the most compelling results came from multivariable analyses that considered emotional exhaustion and depersonalization as the outcome. In these analyses, physical activity appeared to be associated with a reduction in critical conditions. However, it is still unclear whether this association depends on the type, intensity, duration, or frequency of physical activity, as previously mentioned. Some of the included studies (2/21, 10%) suggested that exercising for at least one day per week is sufficient to see a positive effect [58,69], whereas others (2/21, 10%) suggested a frequency of 3 days per week [50,68]. Lebensohn et al [59] observed that, the more frequently HCWs engaged in physical activity, the greater the positive effect.

It is worth noting that, in a few of the studies that considered a multivariable analysis (2/15, 13%), physical activity was not included among the predictors. However, this can sometimes be related to strict variable selection methods.

One limitation of most of the included studies (18/21, 86%) was related to the modality of physical activity measurement. Future studies should consider using objective measurement tools such as pedometers or validated questionnaires such as the International Physical Activity Questionnaire [79] even in its short version [84]. These approaches enhance the accuracy and reliability of data collection. On the other hand, relying on simple questions about regular physical activity or adherence to recommendations for a healthy lifestyle, although suitable for preliminary exploratory investigations, limits the comparability of studies.

The use of precise and detailed definitions to collect measures of intensity and frequency can prove to be a valuable strategy to delve deeper into the dose-response relationship.

### Limitations

Our systematic review has some limitations that warrant consideration. First, our search was confined to 3 databases and only considered English-language articles. Moreover, we did not perform a rerun of the search shortly before submission. However, to mitigate the risk of overlooking relevant papers, we used forward and backward citation tracking, including the use of Google Scholar.

Another potential limitation arises from our eligibility criteria as we considered only HCWs in direct contact with patients and excluded practices with only a light component of physical exercise.

Finally, the heterogeneity in measurement methods and statistical analyses, which we have extensively covered in the Results section, made a meta-analysis inappropriate and precluded the determination of any pooled effect size.

### Conclusions

Our comprehensive overview of studies exploring the association between physical activity and burnout in HCWs revealed a significant level of heterogeneity in definitions, measurements, and analyses adopted in the literature. Our work aimed to advance effective public health practices by addressing this critical issue in the existing evidence. It is important to adopt a clear definition of burnout and physical activity and make thoughtful choices regarding measurement tools and methodologies for data analysis. This becomes particularly crucial when considering that burnout is not a diagnosable disease but rather a multifaceted psychological syndrome that emerges in response to chronic interpersonal stressors in the workplace.

Our findings strongly emphasize the beneficial connection between physical activity and burnout when a statistically significant association is present in the analyses. However, they also highlight the importance of a more in-depth investigation of the specific dependencies on exercise type, intensity, duration, and frequency, knowledge that currently represents a research gap in the field of burnout studies. Moreover, our considerations regarding the measurement of burnout and the comprehensive list of associated factors have the potential to enhance the quality of future studies. Our findings have significant implications for policy makers and health care professionals, underlining the importance of promoting physical activity as an easily accessible mitigation strategy for the well-being of the workforce and the overall effectiveness of the health care system.

## Appendix

Multimedia Appendix 1 Eligibility criteria.

Multimedia Appendix 2 Search strings.

Multimedia Appendix 3 Classification and interpretation of burnout scores.

Multimedia Appendix 4 Predictors adopted in bivariate analyses and in multivariable regression analyses (other than physical activity).

Multimedia Appendix 5 Quality assessment.

Multimedia Appendix 6 PRISMA checklist.

Multimedia Appendix 7 PRISMA-S Checklist.

## Abbreviations

HCW: health care worker
IPAQ-SF: International Physical Activity Questionnaire–Short Form
MBI: Maslach Burnout Inventory
PEO: population, exposure, and outcome
PF: Professional Fulfillment
PRISMA: Preferred Reporting Items for Systematic Reviews and Meta-Analyses
PRISMA-S: Preferred Reporting Items for Systematic Reviews and Meta-Analyses literature search extension
WE: Work Exhaustion

## Data Availability

All data generated or analyzed during this study are included in this published paper (and its supplementary information files).
