# Supplementary material for: Association Between Physical Activity and the Risk of Burnout in Health Care Workers: Systematic Review
Source: JMIR Public Health Surveill. 2024 Mar 18;10:e49772. doi: 10.2196/49772 (PMC10985610; doi:10.2196/49772)
Supplement: Multimedia Appendix 1 [file publichealth_v10i1e49772_app1.docx]

**Association between physical activity and the risk of burnout in healthcare workers: systematic review**

***Multimedia Appendix 1***

**Table S1: Eligibility criteria**

| **Parameter** | **Inclusion criteria** | **Exclusion criteria** |
| --- | --- | --- |
| Population | HCW | Personnel not directly engaged in patient care |
| Exposure | Qualitative or quantitative measures of PhA | PhA not distinct from other supportive, mindfulness strategies or meditation practices with only a light-intensity component of physical activity (yoga or qigong).  PhA measured only through a simple question without any reference to exercise frequency |
| Outcome | Adoption of an assessment tool specifically validated for BO | Absence of a quantitative evaluation of the association between PhA and BO |
| Study setting | No specification (i.e., all types of health setting) | No restriction |
| Study type | No specification (i.e., all types of study) | Literature reviews, grey literature, conference proceedings and unpublished material |
| Language | English |  |
| Publication period | No specification (i.e., all periods of publication) | No restriction |

**Legend**

BO Burnout

HCW Healthcare worker

PhA Physical activity
