# Supplementary material for: Association Between Physical Activity and the Risk of Burnout in Health Care Workers: Systematic Review
Source: JMIR Public Health Surveill. 2024 Mar 18;10:e49772. doi: 10.2196/49772 (PMC10985610; doi:10.2196/49772)
Supplement: Multimedia Appendix 2 [file publichealth_v10i1e49772_app2.docx]

**Association between physical activity and the risk of burnout in healthcare workers: systematic review**

***Supplemental Material – Search Strings***

Ovid MEDLINE(R) and Epub Ahead of Print, In-Process, In-Data-Review & Other Non-Indexed Citations, Daily and Versions <1946 to July 07, 2022>

| **#** | **Search** | **Results** |
| --- | --- | --- |
| 1 | (Healthcare Professional* or Health Care Provider* or Health Personnel or Healthcare Worker* or Nurse* or Nursing Aide* or Nursing Auxiliar* or Nursing Staff or Physician* or Doctor* or practitioner* or clinician* or medic* intern* or medical staff or resident* or medical attendant* or attending physician* or Physical Therapist* or Physiotherapist* or Occupational Therapist* or Midwife or Midwives).mp. or exp Medical Staff, Hospital/ or exp Physicians/ or exp nurse practitioners/ or exp nurse specialists/ or Nurses, Community Health/ or Nurses, Public Health/ or nurse/ | 1822236 |
| 2 | (allergist* or anesthesiologist* or cardiologist* or dermatologist* or endocrinologist* or gastroenterologist* or general practitioner* or GPs or Geriatricians*OR Gerontologist* or gynecologist* or hematologist* or hepatologist* or hospitalist* or immunologist* or internist* or nephrologist* or neonatologist* or neurologist* or obstetrician* or Occupational Health Physician* or oncologist* or ophthalmologist* or Otologist* or otorhinolaryngologist* or pathologist* or pediatric* or physiatrist* or podiatrist* or psychiatrist* or pulmonologist* or radiologist* or respirologist* or rheumatologist* or neurosurgeon* or surgeon or urologist*).ti. | 256148 |
| 3 | patient*.ti. | 2128101 |
| 4 | (1 or 2) not 3 | 1764509 |
| 5 | (Physical Activity or Exercise or Strength Training or Resistance Training or Strengthening Program or Power Training or Bodyweight Training or Endurance Training or Interval Training or High-Intensity Interval Training or Physical Training or Plyometric Drills or Plyometric training or Sprint Interval Training or Gymnastic* or Aerobic* or Fitness Program or Sports).mp. or exp exercise/ | 675393 |
| 6 | (((Swimming or Canoeing or Cycling or Bicycling or Zumba or Bokwa or Aqua Training or Badminton or baseball or basketball or boxing or cricket or exergaming or football or hockey or Jogging or Kayaking or Lacrosse or martial arts or mountaineering or netball* or racquet or rowing or running or rugby or Softball* or Surf* or tennis or soccer or skat* or skiing or track) and field) or volleyball or walking or Water Polo or Weight Lifting or wrestling or Hap Ki Do or HapKiDo or Judo or Karate or Jujitsu or Tae Kwon Do or Aikido or Wushu or Kung Fu or Gong Fu or gongfu or Tai ji or Tai Chi or Ji Quan or taiji*).mp. or exp sports/ or dancing/ or (danc* or ballet*).mp. or Exercise Movement Techniques/ or pilates.mp. | 367573 |
| 7 | 5 or 6 | 859675 |
| 8 | ((burn*3 adj out) or burnout* or depersonali* or emotional exhaustion or physical exhaust*).mp. or burnout, professional/ or burnout, Psychological/ | 27289 |
| 9 | Mental health/ or depression/ or Occupational Stress/ or (mental health or depressi* or Occupation* Stress* or job stress* or job related stress* or work related stress* or workplace stress* or work place stress* or professional stress*).mp. or ((psychological load* or strain* or burden* or stress* or distress* or depress* or anxiet*) adj1 (work* or job* or occupation*)).mp. | 726720 |
| 10 | (AMBQ or CBB or HBI or MBI* or PhBQ or SMBQ or UBOS or Mini Z or BM or PBI or OLBI or BAT CBI or CSFT or HBI or CESQT).mp. | 40292 |
| 11 | 8 or 9 or 10 | 784232 |
| 12 | 4 and 7 and 11 | 4185 |
| 13 | limit 12 to (english language and journal article) | 3900 |
| 14 | limit 13 to (meta analysis or “review” or “systematic review” or “biography” or “case reports” or “clinical conference” or “clinical trial protocol” or “consensus development conference” or “guideline” or “comment” or “patient education handout” or “narrative” or “personal narrative” or “retracted publications” or “published erratum” or “video-audio media”) | 666 |
| 15 | 13 not 14 | 3234 |

Line #10 is based on the following works: Rotenstein and colleagues [1], Edu-Valsania and colleagues [2] and Shoman and colleagues [3]:

And consider the following tools: We considered the following tools: Astudillo and Mendinueta Burnout Questionnaire (AMBQ); Brief Burnout Questionnaire Revised for nursing staff; Burnout Assessment Tool (BAT); Burnout Clinical Subtypes Questionnaire (BCSQ-36/12); Burnout Brief Questionnaire (CBB); Copenhagen Burnout Inventory (CBI); Questionnaire for the Evaluation Work-Related Burnout Syndrome (CESQT); Compassion Satisfaction and Fatigue Test (CSFT); Hamburg Burnout Inventory (HBI); Maslach Burnout Inventory (MBI); MBI–General Survey (MBI-GS); MBI–Human Services Survey (MBI-HSS); OLdenburg Burnout Inventory (OLBI); Physician Burnout Questionnaire (PhBQ); Pines and Aronson Burnout Measure (BM); Psychologist Burnout Inventory (PBI); Rohland et al single-item measure of self-perceived burnout; Shirom–Melamed Burnout Questionnaire (SMBQ); Utrechtse Burnout Schaal (UBOS), i.e. a Dutch adaptation of the MBI; Zero Burnout Program Survey (Mini Z).

SCOPUS <1788 to July 07, 2022>

(

(

TITLE-ABS-KEY ( ”Healthcare Professional*” )  OR  TITLE-ABS-KEY ( ”Health Care Provider*” )  OR  TITLE-ABS-KEY ( ”Health Personnel” )  OR  TITLE-ABS-KEY ( ”Healthcare worker*” )  OR  TITLE-ABS-KEY ( nurse* )  OR  TITLE-ABS-KEY ( ”Nursing Aide*” )  OR  TITLE-ABS-KEY ( ”Nursing Auxiliar*” )  OR  TITLE-ABS-KEY ( ”Nursing Staff” )  OR  TITLE-ABS-KEY ( physician* )  OR  TITLE-ABS-KEY ( doctor* )  OR  TITLE-ABS-KEY ( practitioner* )  OR  TITLE-ABS-KEY ( clinician* )  OR  TITLE-ABS-KEY ( ”medic* intern*” )  OR  TITLE-ABS-KEY ( ”medical staff” )  OR  TITLE-ABS-KEY ( resident* )  OR  TITLE-ABS-KEY ( ”medical attendant*” )  OR  TITLE-ABS-KEY ( ”attending physician*” )  OR  TITLE-ABS-KEY ( ”Physical Therapist*” )  OR  TITLE-ABS-KEY ( physiotherapist* )  OR  TITLE-ABS-KEY ( ”Occupational Therapist*” )  OR  TITLE-ABS-KEY ( midwife )  OR  TITLE-ABS-KEY ( midwives )

)

OR

(

TITLE ( allergist* )  OR  TITLE ( anesthesiologist* )  OR  TITLE ( cardiologist* )  OR  TITLE ( dermatologist* )  OR  TITLE ( endocrinologist* )  OR  TITLE ( gastroenterologist* )  OR  TITLE ( ”general practitioner*” )  OR  TITLE ( gps )  OR  TITLE ( geriatricians )  OR  TITLE ( gerontologist* )  OR  TITLE ( gynecologist* )  OR  TITLE ( hematologist* )  OR  TITLE ( hepatologist* )  OR  TITLE ( hospitalist* )  OR  TITLE ( immunologist* )  OR  TITLE ( internist* )  OR  TITLE ( nephrologist* )  OR  TITLE ( neonatologist* )  OR  TITLE ( neurologist* )  OR  TITLE ( obstetrician* )  OR  TITLE ( ”Occupational Health Physician*” )  OR  TITLE ( oncologist* )  OR  TITLE ( ophthalmologist* )  OR  TITLE ( otologist* )  OR  TITLE ( otorhinolaryngologist* )  OR  TITLE ( pathologist* )  OR  TITLE ( pediatric* )  OR  TITLE ( physiatrist* )  OR  TITLE ( podiatrist* )  OR  TITLE ( psychiatrist* )  OR  TITLE ( pulmonologist* )  OR  TITLE ( radiologist* )  OR  TITLE ( respirologist* )  OR  TITLE ( rheumatologist* )  OR  TITLE ( neurosurgeon* )  OR  TITLE ( surgeon )  OR  TITLE ( urologist* )

)

AND

NOT

TITLE ( patient* )

)

AND

(

(

TITLE-ABS-KEY ( ”Physical Activity” )  OR  TITLE-ABS-KEY ( exercise )  OR  TITLE-ABS-KEY ( ”Strength Training” )  OR  TITLE-ABS-KEY ( ”Resistance Training” )  OR  TITLE-ABS-KEY ( ”Strengthening Program” )  OR  TITLE-ABS-KEY ( ”Power Training” )  OR  TITLE-ABS-KEY ( ”Bodyweight Training” )  OR  TITLE-ABS-KEY ( ”Endurance Training” )  OR  TITLE-ABS-KEY ( ”Interval Training” )  OR  TITLE-ABS-KEY ( ”High-Intensity Interval Training” )  OR  TITLE-ABS-KEY ( ”Physical Training” )  OR  TITLE-ABS-KEY ( ”Plyometric Drills” )  OR  TITLE-ABS-KEY ( ”Plyometric training” )  OR  TITLE-ABS-KEY ( ”Sprint Interval Training” )  OR  TITLE-ABS-KEY ( gymnastic* )  OR  TITLE-ABS-KEY ( aerobic* )  OR  TITLE-ABS-KEY ( ”Fitness Program” )  OR  TITLE-ABS-KEY ( sports )

)

OR

(

TITLE-ABS-KEY ( swimming )  OR  TITLE-ABS-KEY ( canoeing )  OR  TITLE-ABS-KEY ( cycling )  OR  TITLE-ABS-KEY ( bicycling )  OR  TITLE-ABS-KEY ( zumba )  OR  TITLE-ABS-KEY ( bokwa )  OR  TITLE-ABS-KEY ( ”Aqua Training” )  OR  TITLE-ABS-KEY ( badminton )  OR  TITLE-ABS-KEY ( baseball )  OR  TITLE-ABS-KEY ( basketball )  OR  TITLE-ABS-KEY ( boxing )  OR  TITLE-ABS-KEY ( cricket )  OR  TITLE-ABS-KEY ( exergaming )  OR  TITLE-ABS-KEY ( football )  OR  TITLE-ABS-KEY ( hockey )  OR  TITLE-ABS-KEY ( jogging )  OR  TITLE-ABS-KEY ( kayaking )  OR  TITLE-ABS-KEY ( lacrosse )  OR  TITLE-ABS-KEY ( ”martial arts” )  OR  TITLE-ABS-KEY ( mountaineering )  OR  TITLE-ABS-KEY ( netball* )  OR  TITLE-ABS-KEY ( racquet )  OR  TITLE-ABS-KEY ( rowing )  OR  TITLE-ABS-KEY ( running )  OR  TITLE-ABS-KEY ( rugby )  OR  TITLE-ABS-KEY ( softball* )  OR  TITLE-ABS-KEY ( surf )  OR  TITLE-ABS-KEY ( tennis )  OR  TITLE-ABS-KEY ( soccer )  OR  TITLE-ABS-KEY ( skat* )  OR  TITLE-ABS-KEY ( skiing )  OR  TITLE-ABS-KEY ( ”track and field” )  OR  TITLE-ABS-KEY ( volleyball )  OR  TITLE-ABS-KEY ( walking )  OR  TITLE-ABS-KEY ( ”Water Polo” )  OR  TITLE-ABS-KEY ( ”Weight Lifting” )  OR  TITLE-ABS-KEY ( wrestling )  OR  TITLE-ABS-KEY ( ”Hap Ki Do” )  OR  TITLE-ABS-KEY ( ”HapKiDo” )  OR  TITLE-ABS-KEY ( judo )  OR  TITLE-ABS-KEY ( karate )  OR  TITLE-ABS-KEY ( jujitsu )  OR  TITLE-ABS-KEY ( ”Tae Kwon Do” )  OR  TITLE-ABS-KEY ( aikido )  OR  TITLE-ABS-KEY ( wushu )  OR  TITLE-ABS-KEY ( ”Kung Fu” )  OR  TITLE-ABS-KEY ( ”Gong Fu” )  OR  TITLE-ABS-KEY ( gongfu )  OR  TITLE-ABS-KEY ( ”Tai ji” )  OR  TITLE-ABS-KEY ( ”Tai Chi” )  OR  TITLE-ABS-KEY ( ”Ji Quan” )  OR  TITLE-ABS-KEY ( taiji* ) OR  TITLE-ABS-KEY ( danc* ) OR  TITLE-ABS-KEY ( ballet* )  OR  TITLE-ABS-KEY ( pilates )

)

)

AND

(

(

TITLE-ABS-KEY ( burnout* )  OR  TITLE-ABS-KEY ( depersonali* )  OR  TITLE-ABS-KEY ( ”emotional exhaustion” )  OR  TITLE-ABS-KEY ( ”physical exhaust*” )

)

OR

(

TITLE-ABS-KEY ( ”mental health” )  OR  TITLE-ABS-KEY ( depressi* )  OR  TITLE-ABS-KEY ( ”Occupation* Stress*” )  OR  TITLE-ABS-KEY ( ”job stress*” )  OR  TITLE-ABS-KEY ( ”job related stress*” )  OR  TITLE-ABS-KEY ( ”work related stress*” )  OR  TITLE-ABS-KEY ( ”workplace stress*” )  OR  TITLE-ABS-KEY ( ”work place stress*” )  OR  TITLE-ABS-KEY ( ”professional stress*” )

)

OR

(

TITLE-ABS-KEY ( AMBQ )  OR   TITLE-ABS-KEY ( CBB )  OR   TITLE-ABS-KEY ( HBI )  OR   TITLE-ABS-KEY ( PhBQ )  OR   TITLE-ABS-KEY ( SMBQ )  OR   TITLE-ABS-KEY ( UBOS )  OR   TITLE-ABS-KEY ( Mini Z )  OR   TITLE-ABS-KEY ( mbi* )  OR  TITLE-ABS-KEY ( cbi )  OR  TITLE-ABS-KEY ( BM )  OR  TITLE-ABS-KEY ( PBI )  OR  TITLE-ABS-KEY ( OLBI )   OR  TITLE-ABS-KEY ( csft )  OR  TITLE-ABS-KEY ( hbi )  OR  TITLE-ABS-KEY ( cesqt )

)

)

AND

(

LIMIT-TO ( DOCTYPE ,  ”ar” )

)

AND

(

LIMIT-TO ( LANGUAGE ,  ”English” )

)

AND

(

EXCLUDE ( SUBJAREA ,  ”BIOC” )  OR  EXCLUDE ( SUBJAREA ,  ”ENVI” )  OR  EXCLUDE ( SUBJAREA ,  ”PHAR” )  OR  EXCLUDE ( SUBJAREA ,  ”AGRI” )  OR  EXCLUDE ( SUBJAREA ,  ”ARTS” )  OR  EXCLUDE ( SUBJAREA ,  ”ENGI” )  OR  EXCLUDE ( SUBJAREA ,  ”BUSI” )  OR  EXCLUDE ( SUBJAREA ,  ”IMMU” )  OR  EXCLUDE ( SUBJAREA ,  ”COMP” )  OR  EXCLUDE ( SUBJAREA ,  ”MATH” )  OR  EXCLUDE ( SUBJAREA ,  ”ENER” )  OR  EXCLUDE ( SUBJAREA ,  ”ECON” )  OR  EXCLUDE ( SUBJAREA ,  ”VETE” )  OR  EXCLUDE ( SUBJAREA ,  ”CENG” )  OR  EXCLUDE ( SUBJAREA ,  ”EART” )  OR  EXCLUDE ( SUBJAREA ,  ”PHYS” )  OR  EXCLUDE ( SUBJAREA ,  ”CHEM” )  OR  EXCLUDE ( SUBJAREA ,  ”DECI” )  OR  EXCLUDE ( SUBJAREA ,  ”MATE” )

)

5,570 documents found

*Search string in PsychInfo*

(

(

**Any Field**: (“Healthcare Professional*”)  *OR*  **Any Field**: (“Health Care Provider*”)  *OR*  **Any Field**: (“Health Personnel”)  *OR*  **Any Field**: (“Healthcare Worker*”)  *OR*  **Any Field**: (Nurse*)  *OR*  **Any Field**: (“Nursing Aide*”)  *OR*  **Any Field**: (“Nursing Auxiliar*”)  *OR*  **Any Field**: (“Nursing Staff”)  *OR*  **Any Field**: (Physician*)  *OR*  **Any Field**: (Doctor*)  *OR***Any Field**: (practitioner*)  *OR*  **Any Field**: (clinician*) *OR*  **Any Field**: (“medic* intern*”)  *OR*  **Any Field**: (“medical staff”)  *OR*  **Any Field**: (resident*)  *OR*  **Any Field**: (“medical attendant*”)  *OR*  **Any Field**: (“attending physician*”)  *OR*  **Any Field**: (“Physical Therapist*”)  *OR*  **Any Field**: (Physiotherapist*)  *OR* **Any Field**: (“Occupational Therapist*”)  *OR*  **Any Field**: (Midwife)  *OR*  **Any Field**: (Midwives)

)

*OR*

(

**IndexTermsFilt**: (“Physicians”)  *OR* **IndexTermsFilt**: (“clinician”)  *OR*  **IndexTermsFilt**: (“nurses”)  *OR*  **IndexTermsFilt**: (“Public Health Service Nurses”)

)

)

*AND*

(

(

**Any Field**: (“Physical Activity”)  *OR*  **Any Field**: (Exercise)  *OR*  **Any Field**: (“Strength Training”)  *OR*  **Any Field**: (“Resistance Training”)  *OR*  **Any Field**: (“Strengthening Program”)  *OR*  **Any Field**: (“Power Training”)  *OR* **Any Field**: (“Bodyweight Training”)  *OR*  **Any Field**: (“Endurance Training”)  *OR*  **Any Field**: (“Interval Training”)  *OR*  **Any Field**: (“High-Intensity Interval Training”)  *OR*  **Any Field**: (“Physical Training”)  *OR* **Any Field**: (“Plyometric Drills”)  *OR*  **Any Field**: (“Plyometric training”)  *OR*  **Any Field**: (“Sprint Interval Training”)  *OR*  **Any Field**: (Gymnastic*)  *OR* **Any Field**: (Aerobic*)  *OR*  **Any Field**: (“Fitness Program”)  *OR*  **Any Field**: (Sports)

)

*OR*

(

**IndexTermsFilt**: (“Sports”)  *OR*  **IndexTermsFilt**: (“Dance”)  *OR*  **IndexTermsFilt**: (“Athletic Training”) *OR*  **IndexTermsFilt**: (“Exercise”)  *OR* **IndexTermsFilt**: (“Aerobic Exercise”)

)

)

*AND*

(

(

**Any Field**: (burn-out)  *OR*  **Any Field**: (burnout*)  *OR*  **Any Field**: (depersonali*)  *OR*  **Any Field**: (“emotional exhaustion”)  *OR*  **Any Field**: (“physical exhaust*”)

)

*OR*

(

**IndexTermsFilt**: (“burnout”)  *OR* **IndexTermsFilt**: (“Occupational Stress”)  *OR* **IndexTermsFilt**: (“Mental health”)  *OR* **IndexTermsFilt**: (“Depression (Emotion)”)  *OR* **IndexTermsFilt**: (“Reactive Depression”)

)

*OR*

(

**Any Field**: (“mental health”)  *OR*  **Any Field**: (depressi*)  *OR*  **Any Field**: (“Occupation* Stress*”) *OR*  **Any Field**: (“job stress*”)  *OR*  **Any Field**: (“job related stress*”)  *OR*  **Any Field**: (“work related stress*”)  *OR*  **Any Field**: (“workplace stress*”)  *OR* **Any Field**: (“work place stress*”)  *OR*  **Any Field**: (“professional stress*”)

)

)

*AND*

**Document Type**: Journal Article

129 documents found

References

1. Rotenstein, L.S.; Torre, M.; Ramos, M.A.; Rosales, R.C.; Guille, C.; Sen, S.; Mata, D.A. Prevalence of Burnout Among Physicians: A Systematic Review. JAMA 2018, 320, 1131–1150, doi:10.1001/jama.2018.12777.

2. Edu-Valsania, S.; Laguia, A.; Moriano, J.A. Burnout: A Review of Theory and Measurement. Int. J. Environ. Res. Public Health 2022, 19, doi:10.3390/ijerph19031780.

3. Shoman, Y.; Marca, S.C.; Bianchi, R.; Godderis, L.; van der Molen, H.F.; Guseva Canu, I. Psychometric properties of burnout measures: a systematic review. Epidemiol. Psychiatr. Sci. 2021, 30, e8, doi:10.1017/S2045796020001134.
