# Supplementary material for: Association Between Physical Activity and the Risk of Burnout in Health Care Workers: Systematic Review
Source: JMIR Public Health Surveill. 2024 Mar 18;10:e49772. doi: 10.2196/49772 (PMC10985610; doi:10.2196/49772)
Supplement: Multimedia Appendix 3 [file publichealth_v10i1e49772_app3.docx]

**Association between physical activity and the risk of burnout in healthcare workers: systematic review**

***Multimedia Appendix 3***

**Table S1: Classification and interpretation of burnout scores**

| **Method** | **Categories** | **Ranges of score in each dimension** | | | **Studies adopting the categorization** |
| --- | --- | --- | --- | --- | --- |
| **MBI** |  | **EE** | **DP** | **PA** |  |
| 22-item MBI Maslach 1996 [72] –  *Normative data for Medicine* | High Moderate Low | ≥27 19-26 ≤18 | ≥10 6-9 ≤5 | ≥40 34-39 ≤33 | [52,53,62,66,69] and [59,60]a |
| 22-item MBI Maslach 1996 [72] –  *Scoring key* | High Moderate Low | ≥27 17-26 ≤16 | ≥13 7-12 ≤6 | ≥39 32-38 ≤31 | [51,55,58,67,68] |
| 22-item MBI Ajab 2021 | High Moderate Low | ≥38 19-37 ≤18 | ≥21 11-20 ≤10 | ≥34 17-33 ≤16 | [49] |
| 22-item MBI Ghoraishian 2022 | High Moderate Low | ≥30 18-29 ≤17 | ≥12 7-11 ≤6 | ≥40 34-39 ≤33 | [56] |
| 9-item MBI (aMBI) Panse N., 2020 | High Low | ≥10 ≤9 | ≥10 ≤9 | ≥10 ≤9 | [63] |
|  |  |  |  |  |  |
| **CBI** |  | **PeE** | **WrE** | **PaE** |  |
| 19-item CBI [73] | High Low | ≥50 <50 [0-100] | ≥50 <50 [0-100] | ≥50 <50 [0-100] | [61] |

**Legend**

The *Reference* column provides information about the derivation or validation studies in which the *Ranges of score* are initially reported. If authors referred no validation study, we assumed this as an arbitrary choice and directly reported the adopting study (hence the same as in *Studies adopting the categorization*). The full range of score is [0-132], if not otherwise reported. Red ranges indicate a critical condition.

a Authors interpretation

DP Depersonalization

EE Emotional Exhaustion

MBI Maslach Burnout Inventory

PA Personal accomplishment

PaE Patient-related physical and psychological Exhaustion

PeE Personal physical and psychological Exhaustion

WrE Work-related physical and psychological Exhaustion
