# Supplementary material for: Association Between Physical Activity and the Risk of Burnout in Health Care Workers: Systematic Review
Source: JMIR Public Health Surveill. 2024 Mar 18;10:e49772. doi: 10.2196/49772 (PMC10985610; doi:10.2196/49772)
Supplement: Multimedia Appendix 5 [file publichealth_v10i1e49772_app5.docx]

***Association between physical activity and the risk of burnout in healthcare workers: systematic review***

**Table S1: Quality assessment**

| **Study** | **1 - Were the criteria for inclusion in the sample clearly defined?** | **2 - Were the study subjects and the setting described in detail?** | **3 - Was the exposure measured in a valid and reliable way?** | **4 - Were objective, standard criteria used for measurement of the condition?** | **5 - Were confounding factors identified?** | **6 - Were strategies to deal with confounding factors stated?** | **7 - Were the outcomes measured in a valid and reliable way?** | **8 - Was appropriate statistical analysis used?** |
| --- | --- | --- | --- | --- | --- | --- | --- | --- |
| Ajab S, 2021 [49] | Yes | Yes | Partial | Yes | na | na | Yes | Unclear |
| Alvares MEM, 2020 [50] | Yes | Yes | Partial | Yes | na | na | Partial | No |
| Bin Dahmash A, 2020 [51] | Yes | Yes | Partial | Yes | na | na | Partial | Yes |
| Chokri A, 2021 [52] | Yes | Yes | Yes | Yes | na | na | Yes | Yes |
| de la Cruz SP, 2020 [53] | Yes | Yes | Partial | Yes | na | na | Yes | Yes |
| Eckstein J, 2022 [54] | Yes | Partial | Partial | Yes | na | na | Yes | Partial |
| Feng S, 2018 [55] | Yes | Yes | Partial | Yes | na | na | Partial | Partial |
| Ghoraishian M, 2022 [56] | Yes | Yes | Partial | Yes | na | na | No | Partial |
| Goldberg R, 1996 [57] | Yes | Yes | Partial | Yes | na | na | Partial | Yes |
| Hu Z, 2021 [58] | Yes | Yes | Partial | Yes | na | na | Partial | No |
| Lebensohn P, 2013 [59] | Yes | Yes | Partial | Yes | na | na | Yes | Partial |
| McClafferty H, 2021 [60] | Yes | Yes | Yes | Yes | na | na | Yes | Partial |
| Ng APP, 2020 [61] | Yes | Yes | Partial | Yes | na | na | Yes | Partial |
| Olson SM, 2014 [62] | Yes | Yes | Yes | Yes | Partial | No | Partial | Partial |
| Panse N, 2020 [63] | Yes | Yes | Partial | Yes | na | na | No | Partial |
| Reed L, 2020 [64] | Yes | No | Partial | Yes | na | na | Yes | Unclear |
| Shanafelt TD, 2012 [65] | Yes | Yes | Partial | Yes | na | na | Yes | Yes |
| Tiwari V, 2020 [66] | Yes | Yes | Partial | Yes | na | na | Partial | Partial |
| Vinnikov D, 2019 [67] | Yes | Yes | Partial | Yes | Partial | Partial | Partial | Partial |
| Vinnikov D, 2021 [68] | Yes | Yes | Partial | Yes | na | na | Partial | Partial |
| Yang G, 2018 [69] | Yes | Yes | Partial | Yes | Partial | Partial | Partial | Partial |

The quality of studies included in the analysis was assessed using the JBI Critical Appraisal Checklist for cross-sectional studies [45]. This checklist consists of eight items that are rated as “yes”, “no”, “unclear” or “not applicable” (na). To further refine our judgment, we added “partial” as a fifth option.
